# Doctors at the Head of the Army

**Published:** 1910-09

**Authors:** 


### Doctors at the Head of the Army.

LEONARD WOOD, Senior Major General, by virtue of his rank, has become Chief of the General Staff and therefore in actual command of the Army of the United States. This is a condition that could not occur, perhaps, in any other country in the world, certainly not among the great powers.

General Wood's military career has been remarkable. Entering the army as an assistant surgeon, he participated in the frontier or western service for some years, during which he was assigned on more than one occasion, we believe, to the actual command of troops. This, of course, was because of exigencies that arose which line officers were not available to meet; but assistant surgeon Wood was equal to the duties imposed upon him.

The beginning of the war with Spain in 1898, found Dr. Wood still in the medical corps with the rank of Captain, but anxious for field service in the line. He, in company with Theodore Roosevelt, was authorised to raise a regiment of cavalry known as the "Rough Riders," which was assigned to the cavalry division of Major General Joseph Wheeler. At Siboney, the first engagement in Cuba, Colonel Wood was promoted to Brigadier General, holding this rank until 1903, when he became Major General. His service in Cuba during the "reconstruction period" is well known to everybody familiar with the history of that time. General Wood served in the Philippines for some years, and now comes back to us in the health and vigor of a ripe and sturdy manhood. He is due to retire October 9, 1924, hence fourteen years of active service yet remain to engage his attention. He will keep himself busy until the cessation of his military career.

By a singular coincidence the Adjutant General of the Army, Major General Frederick C. Ainsworth, is a physician by education. He also entered the army as an assistant surgeon. His career has been notable and his transfer from the medical corps to the line was well earned. With two such sterling characters directing the affairs of the army, the medical profession may well take pride in its administration.

ATTENTION is invited to the letter of Drs. Lovett and Snow, published elsewhere relating to epidemic poliomyelitis, supposed to be prevalent to greater or less extent in various parts of the United States and Canada. The health authorities, state and local, no doubt, will give the suggestions contained in the letter referred to that consideration which the existing conditions merit.

---

THE Buffalo Department of Health has issued a new form of death certificate in conformity with the requirements of the State Commissioner of Health. This new certificate is to take the place of the one previously in use, and will be accepted alone after this date. It seems that the new form is somewhat cumbersome in its requirements, though we recognise the fact that there is liability to shield criminals by carelessness in giving a certificate of death. No doubt in years gone by too much haste has been permitted in making up this important record. It is a paper intended to last, and should contain accurate scientific information. The slip-shod manner of its preparation by many should not be tolerated, and every physician worthy the name should lend his influence toward the perfection of vital statistics.

---

IT has been observed that flies are less numerous than formerly, and an explanation is offered, that the fumes from the motor cars act as a fly-killer. At all events the following from the *London Mirror,* is printed for what it may be worth in support of this theory.

The air of the London streets is purer at the moment than ever it has been before and for this we have partly to thank the unsightly and noisy motor omnibus. Flies, insects and germs, it appears, are killed in their millions by the petrol fumes of the ever-increasing motor traffic of London.

At a meeting of the Salmon and Trout Association, Lord Desborough said it had been stated that insect life in Parliament Square had been put an end to altogether by the motor traffic. This statement was fully borne out by a well-known London chemist.

"The fumes from motor omnibuses and other petrol-driven vehicles," he said, "are undoubtedly a powerful insecticide. The creosote vapor exuded from the exhaust pipes is not only a fly-killer, but as carbonised matter, it clears the air of germs and other impurities.

"The large number of petrol-driven vehicles in London at the present time, each exuding antiseptic vapor is largely responsible for a big decrease in insect life. Londoners are constantly denouncing this vapor as a nuisance. Really, however, it is beneficial to their health."

A Strand fruiterer said that this year there had been a remarkable absence of flies in London.

It is pleasant to note that, through the efforts of Dr. E. A. Bowerman, police surgeon, two rooms are being fitted up on the second floor of police station No. 3 in Pearl street, for the detention of persons suspected of insanity, who may fall into the hands of the police. One room will be for women and the other for men and these will have every convenience for sleeping and toilet purposes. There are several men on the police force who have been orderlies or otherwise employed at state hospitals, and they have a fair knowledge of how the insane should be dealt with. One of these will be constantly on duty. Dr. Bowerman, himself, served for a number of years as a resident physician at the Buffalo State Hospital, and therefore is familiar with the care of the insane. To its shame Buffalo heretofore has been without detention quarters of this nature, and Dr. Bowerman is entitled to the thanks of every right minded citizen for instituting this humane system.

---

The Department of Health of Buffalo, according to statements made by the newspaper press, will give object lessons in sanitation and preventive medicine, through the medium of moving pictures and lantern slides. The acting health commissioner Dr. Schaefer, is quoted as saying that he is endeavoring to secure, through Genesee street business men, a store or hall where pictures can be exhibited during the carnival which they are preparing for on that street.

The department has a large collection of slides covering typhoid fever, general sanitation and hygiene, milk and its relation to the public health, tuberculosis, and general health problems. When to these slides are added films with moving pictures the exhibit should command a large audience, and prove instructive to those who most need such information.

---

The prevalence of whooping cough in Buffalo is receiving the attention of the Health Department. Drinking fountains, where the thirsty are served with a cup are a menace to health, and serve as spreaders of infectious diseases. Particularly is it so with those diseases to which children are liable, as they are proverbial patrons of drinking places. Recently the chairman of the park board, Mr. Maurice M. Wall, so the story goes, witnessed a very convincing object lesson on this point. Walking in the park with his little daughter a few days ago, the latter approached one of the fountains for a drink. But a boy with whooping cough came first and after drinking was seized with a violent paroxysm of cough. The commissioner hastily took the cup out of the little girl's hands and persuaded her to wait until reaching home to satiate her thirst. It is probable that sanitary drinking fountains will be installed in the park at an early day.

THE prevalence of cholera in some parts of Europe, notably in southern Russia and Italy, need not alarm the inhabitants of this country. The spread of the disease in Europe is due chiefly to the lack of proper sanitary measures,—in short, to filth. Our rigid quarantine will prevent the entrance of the disease to our shores; or, if it should come it will be quickly stamped out by efficient sanitary means. The government already is alert and is taking every precaution to prevent the introduction of cholera here by immigrants.

